# Pembrolizumab‐caused polyradiculoneuropathy as an immune‐related adverse event

**DOI:** 10.1111/neup.12729

**Published:** 2021-05-03

**Authors:** Takashi Kurashige, Mineyo Mito, Hideki Yamamoto, Tomohito Sugiura, Takashi Onoe, Kazuya Kuraoka, Kikuo Nakano, Tsuyoshi Torii

**Affiliations:** ^1^ Department of Neurology National Hospital Organization Kure Medical Center and Chugoku Cancer Center Kure, Hiroshima Japan; ^2^ Department of Respiratory National Hospital Organization Kure Medical Center and Chugoku Cancer Center Kure, Hiroshima Japan; ^3^ Department of Diagnostic Pathology National Hospital Organization Kure Medical Center and Chugoku Cancer Center Kure, Hiroshima Japan; ^4^ Department of Surgery National Hospital Organization Kure Medical Center and Chugoku Cancer Center Kure, Hiroshima Japan

**Keywords:** immune‐checkpoint inhibitor, immune‐related adverse event, subacute sensorimotor neuronopathy, PD‐1

## Abstract

Immune‐related adverse events (irAEs) commonly involve the gastrointestinal tract, endocrine glands, skin, and liver, and rarely the nervous system. The pathomechanism of irAEs in the nervous system is unclear, and so characterizing these severe toxic effects is a priority, even if irAEs are uncommon in the nervous system. Our patient presented subacute muscle weakness and dysesthesia with colitis as irAEs caused by pembrolizumab, one of the anti‐programmed death‐1 (PD‐1) antibodies. Electromyography revealed abundant fibrillations and fasciculations of upper and lower extremities and severe reduction in motor unit potentials; however, antineutrophil cytoplasmic antibodies, rheumatoid factor, autoantibodies against Hu and Yo, and anti‐ganglioside antibodies, such as GQ1b, were undetectable in the serum. Although he was treated with high‐dose glucocorticoids, antibiotics, and a monoclonal anti‐tumor necrosis factor alpha (TNFα) antibody, he developed colonic perforation. The total colorectal resection was performed, and the resected colon showed mucosal defect and perforation. He died of lung aspergillosis. Postmortem examination revealed CD8‐positive lymphocyte infiltration around neurons of dorsal root ganglia. The sciatic nerve displayed the widening of myelin laminae and thinning of myelinated fibers but not a decrease in the density of myelinated nerve fibers. In the sural nerve, the density of myelinated fibers slightly decreased, and some fibers showed less densely myelinated laminae. Drug safety information, including previous randomized trials of anti‐PD‐1 and anti‐cytotoxic T‐lymphocyte–associated antigen‐4 (CTLA‐4) antibodies, showed that patients treated with anti‐PD‐1 antibodies appeared to have more frequent and severe peripheral neuropathies compared to those in patients who received anti‐CTLA‐4 antibodies (1.59% *vs*. 0.69%; Fisher exact test, *P* < 0.001; three severe events *vs*. zero severe events). The present results and drug safety information suggest that the pathomechanism of irAEs caused by anti‐PD‐1 antibodies is different from that by anti‐CTLA‐4 antibodies. The neurological irAEs might be clues to solving the pathomechanism of irAEs.

## INTRODUCTION

Immune checkpoint inhibitors have transformed the treatment of various cancers by releasing restrained anticancerous immune responses.^1^ Anti‐programmed death‐1 (PD‐1) antibodies, including nivolumab and pembrolizumab, and the anti‐cytotoxic T‐lymphocyte–associated antigen‐4 (CTLA‐4) antibody, named ipilimumab have individually improved overall survival for patients with various cancers.^2^ Although any organ system can be affected, immune‐related adverse events (irAEs) most commonly involve the gastrointestinal tract, endocrine glands, skin, and liver.^3^ There is no difference in the incidence of irAEs affecting these organs between anti‐PD‐1 and anti‐CTLA‐4 antibodies. The irAEs involving these organs are all thought to arise from aberrant activation of autoreactive T lymphocytes;^3^, ^4^ the reaction is more frequent and severe when ipilimumab and nivolumab are used in combination.^5^

Peripheral neuropathies in clinical trials with anti‐PD‐1 and anti‐CTLA‐4 antibodies have rarely been reported. The pathomechanism of irAEs in the nervous system is still unclear, although the American Society of Clinical Oncology and National Comprehensive Cancer Network guidelines established recommendations for evaluating and treating irAEs.^6^ Thus, characterizing these severe toxic effects is a priority, even if irAEs are uncommon in the nervous system.

Here, we report for the first time a case of lethal polyradiculoneuropathy in a lung cancer patient treated with pembrolizumab. The patient presented clinically subacute muscle weakness and dysesthesia with colitis as pembrolizumab‐induced irAEs, which were pathologically characterized by CD8‐positive lymphocyte infiltration around neurons in dorsal root ganglia (DRG) and transmural colitis. Our findings suggest that irAEs of the nervous system differ from those of other organs. These findings may imply that a neurological irAE is a clue to solving the difference in the pathomechanism of irAEs between anti‐PD‐1 and anti‐CTLA‐4 antibodies.

## CLINICAL SUMMARY

**Fig 1:**
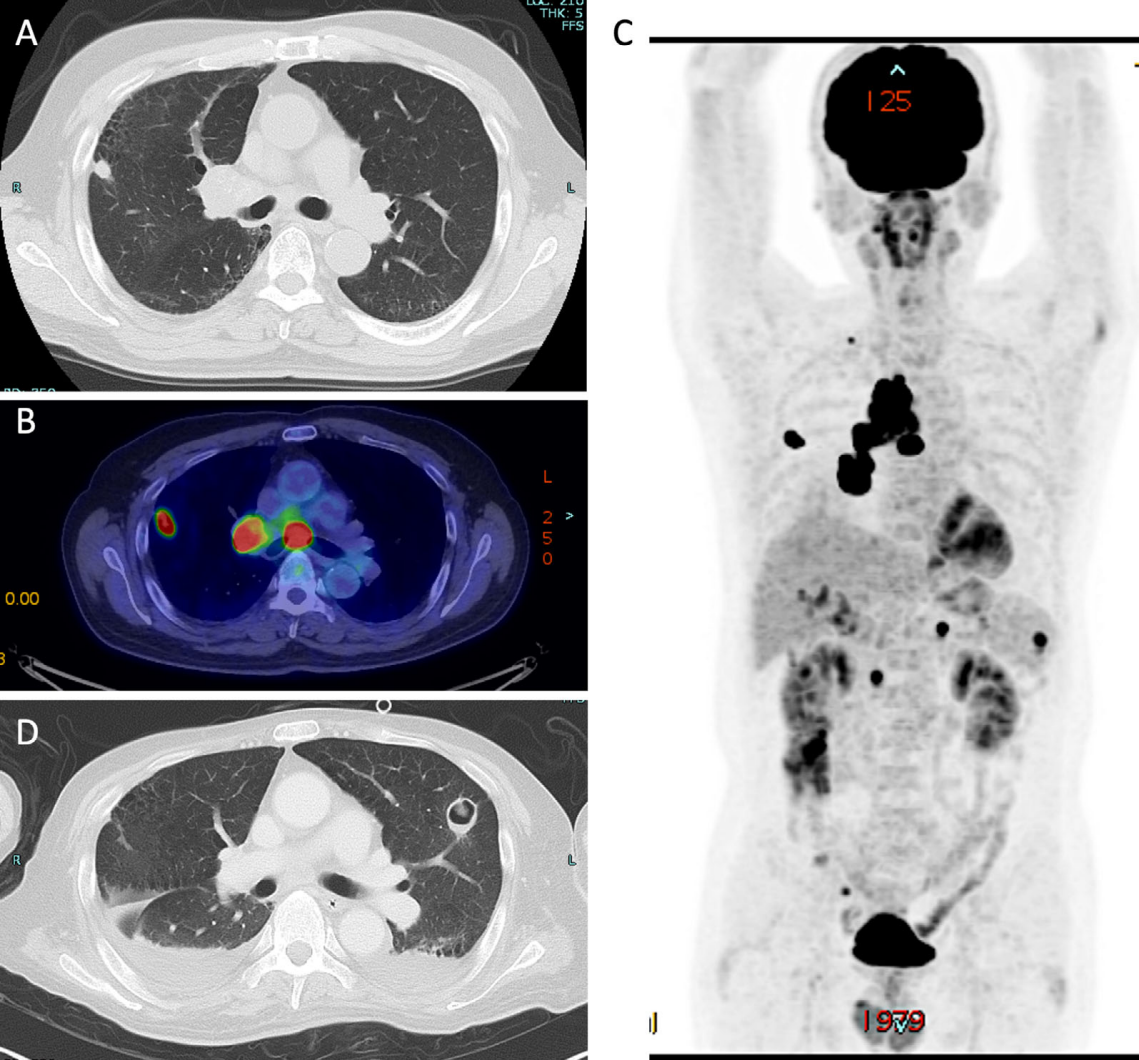
Radiological findings of the lung cancer. (A, D) Chest CT reveals pulmonary opacity in the upper lobe of the right lung three months before admission. (B, C) PET‐CT reveals hot spots in the right lung, lymph nodes of the mediastinum, spleen, and ilium. (D) Chest CT at the patient’s admission reveals a reduction in size of the lung tumor.

A 56‐year‐old man with lung cancer was admitted to the hospital with bloody stool, muscle weakness, and pain in his extremities 20 days after receiving his three‐times treatment of pembrolizumab (200 mg, each). One year before the admission, the patient presented a severe cough, and pulmonary opacity in the upper lobe of the right lung was pointed out at the medical examination five months before the admission (Fig. 1A). Positron emission tomography (PET)‐computed tomography (CT) (PET‐CT) revealed hot spots in the right lung, lymph nodes of the mediastinum, spleen, and ilium (Fig. 1B, C). The mediastinoscopy with biopsy revealed metastasis to the mediastinum lymph nodes based on the patientʼs diagnosis: atypical carcinoid of the lung (T1bN2M1c, cStage IV). The patient was treated with cisplatin and etoposide as the first‐line chemotherapy, but it was not effective for the patient's lung tumor. Pembrolizumab was administered as the second‐line chemotherapy for lung cancer due to the high programmed death‐ligand 1 expression in this tumor with the labeling index of 70%. The patient was treated three times with pembrolizumab, and the size of the lung cancer was reduced radiologically (Fig. 1D).

**Fig 2:**
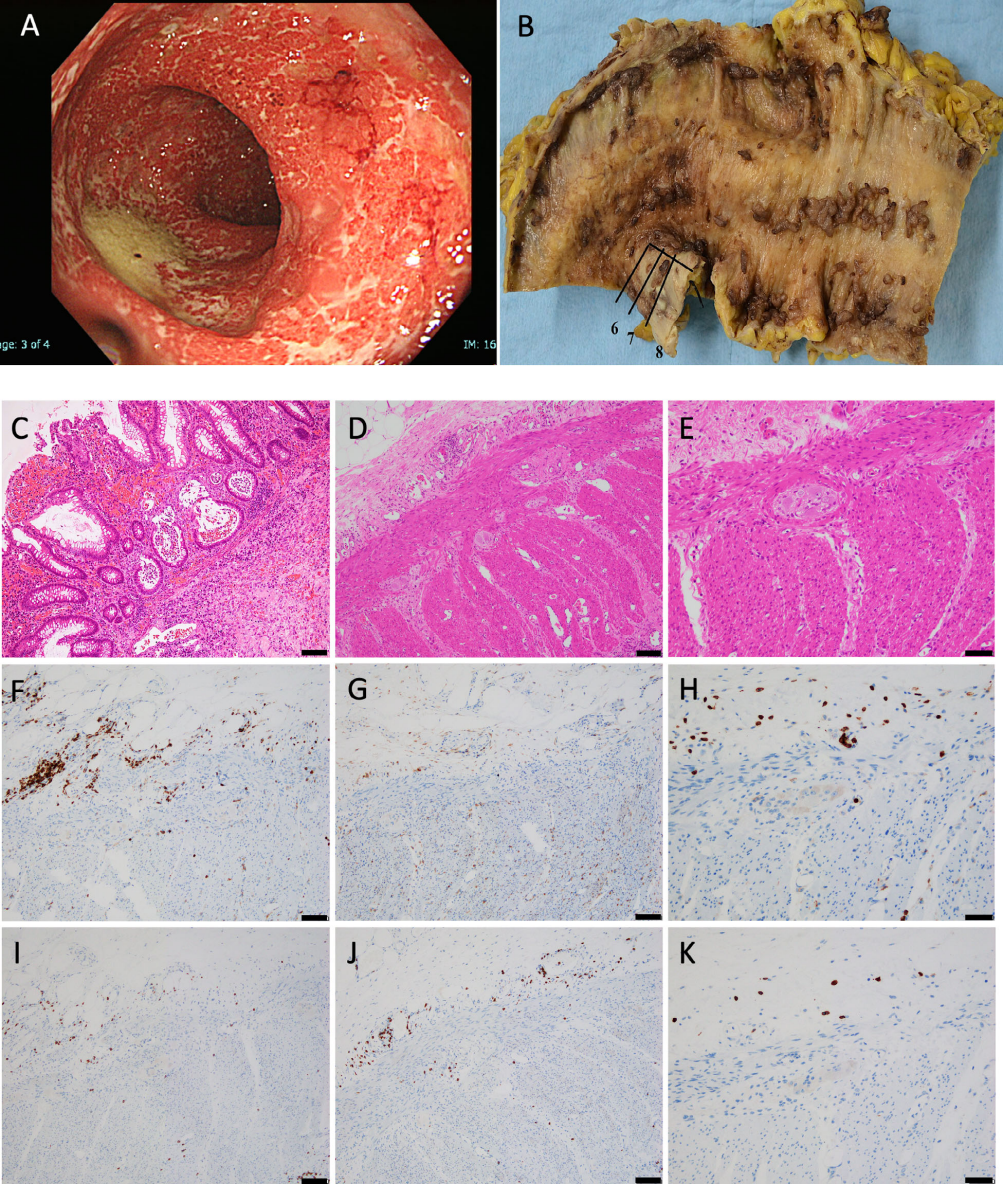
Findings on total colonoscopy (A) and surgical pathology (B‐K) of the colon. (A) A total colonoscopic image shows colitis. (B) The resected colon specimen grossly shows mucosal defect and colon perforation. (C‐E) Histopathological examination on HE‐stained sections of the colon reveals transmural colitis (C) and no significant lymphocytic infiltration in the submucosal plexuses (D, E). (F, G) CD45‐positive cells are observed in all layers of the colon. (H) All of the colonic plexuses show no infiltration of CD45‐positive cells. (I, J) There are scattered lymphocytes positive for CD4 (I) and CD8 (J). (K) No CD8‐positive lymphocytic infiltrates are observed in any colonic plexuses. Immunohistochemical staining (F‐K). Scale bars: 100 μm (C, D, F, G, I, J), 50 μm (E, H, K).

An initial blood test revealed increased leukocyte count and CRP level (9400/μL [normal range, < 8000] and 8.43 IU/L [normal range < 1.00], respectively). Abdominal CT revealed colonic edema. A total colonoscopy revealed colitis (Fig. 2A). Electromyography revealed abundant fibrillations and fasciculations of the upper and lower extremities and severe reduction in motor unit potentials. We could not perform the nerve conduction study because his performance status worsened at that time. The anti‐neutrophil cytoplasmic antibodies, rheumatoid factor, anti‐neuronal autoantibodies against Hu and Yo, and anti‐ganglioside antibodies such as GQ1b were undetectable in the serum. He was treated with high‐dose glucocorticoids (intravenous methylprednisolone administered at 40 mg per day) and antibiotics (intravenous meropenem administration at 2 g per day) for 24 h after admission and monoclonal anti‐tumor necrosis factor alpha (TNFα) antibody (intravenous infliximab administration at 350 mg per day) eight days after admission. He developed colonic perforation 37 days after admission, and a total colorectal resection was performed. Grossly, the resected colon specimen exhibited mucosal defect and perforation (Fig. 2B). Histopathological examination revealed nonspecific inflammation changes characterized by transmural colitis (Fig. 2C) without lymphocytic infiltration in submucosal plexuses (Fig. 2D, E). Immunohistochemical staining revealed that inflammatory cells were positive for CD45 (Fig. 2F–H), CD4 (Fig. 2I), and CD8 (Fig. 2J, K). However, all colonic plexuses observed in these surgical specimens showed the lack of infiltration of inflammatory cells positive for CD45 (Fig. 2F–H), CD4 (Fig. 2I), and CD8 (Fig. 2J, K). After his colorectal resection, his muscle weakness and respiratory failure remained. He died of lung aspergillosis 58 days after admission.

## POSTMORTEM PATHOLOGICAL FINDINGS

**Fig 3:**
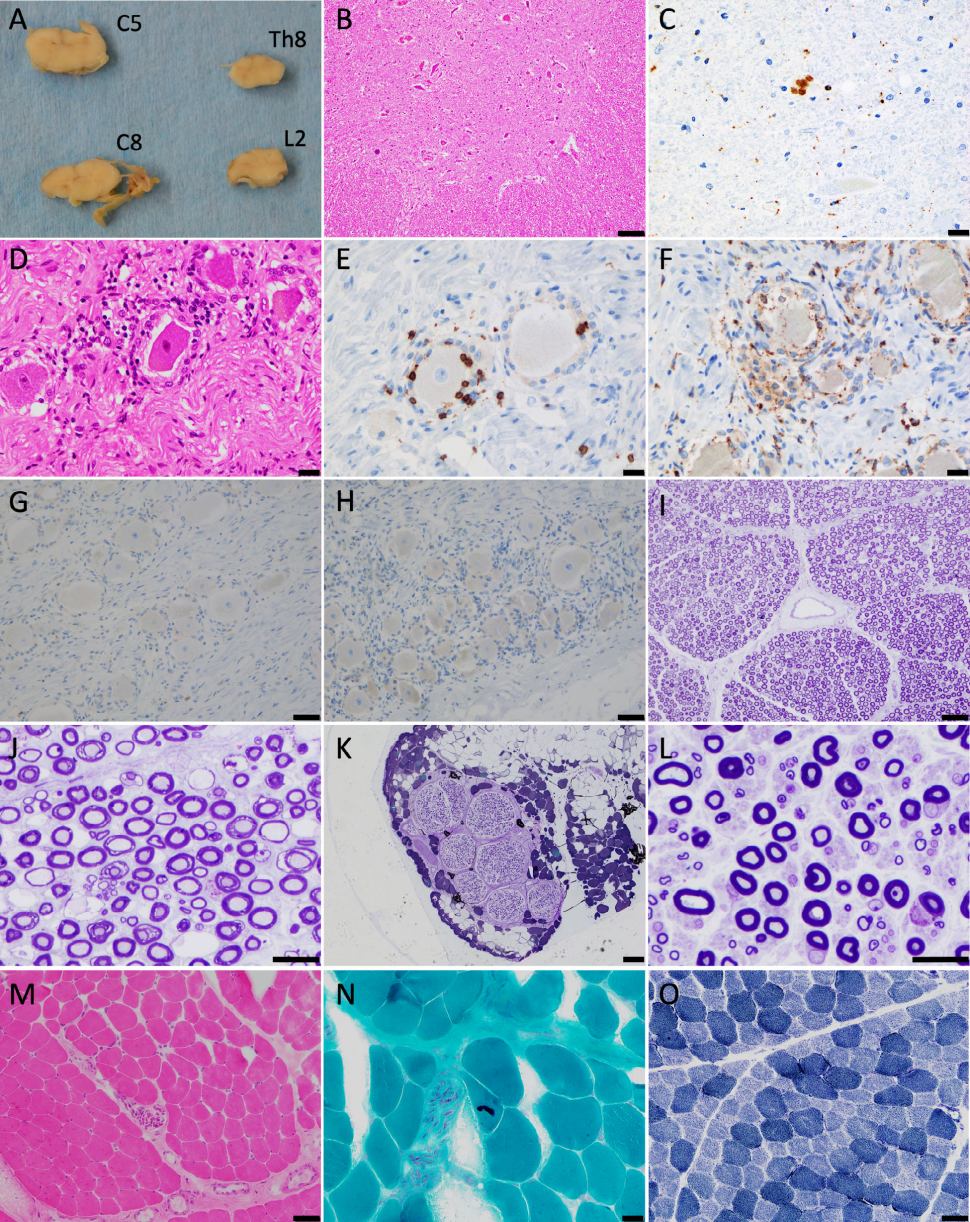
Pathological finding of the spinal cord, dorsal root ganglia, and peripheral nerves. (A) Grossly, the spinal cord does not show any abnormalities. (B) The spinal cord anterior horn neurons show a decrease in number on an HE‐stained section. (C) Immunohistochemically, CD68‐positive macrophage/microglia infiltrates are observed around the spinal cord neurons. (D) The dorsal root ganglia show severe lymphocyte infiltration around the neurons on an HE‐stained section. (E‐H) Infiltrating cells are CD8‐positive T lymphocytes (E) and CD68‐positive macrophages (F) and negative for CD4 (G) and CD20 (H). (I, J) Toluidine blue‐stained Epon‐embedded semithin sections of the sciatic nerve show no significant decrease in the density of myelinated fibers (I) and widening of myelin laminae and thinning of myelinated fibers (J). (K, L) Toluidine blue‐stained semithin sections of the sural nerve show a slight decrease in the density of myelinated fibers (K) and thinning of myelin laminae of some fibers (L). (M) The fibularis brevis muscle shows preserved intramuscular nerve bundles without inflammatory cell infiltration on an HE‐stained section. (N, O) In the the muscle, cytoplasmic bodies are scattered in myofibrils and detected on a modified Gomori‐trichrome‐stained section (N) and preserved intermyofibrillar networks on an NADH‐TR‐stained section (O). Immunohistochemical staining (E‐H). Scale bars: 100 μm (B), 20 μm (C‐F, J, L, N), 50 μm (G‐I, M, O), 200 μm (K).

A postmortem examination of several organs except for the brain was performed 2 h after his death. Gross examination revealed no significant abnormality in the spinal cord (Fig. 3A), DRG, or sciatic or sural nerve. Histopathological observation revealed that neurons in the spinal cord did not decrease (Fig. 3B); instead, they were infiltrated by CD68‐positive macrophage/microglia (Fig. )Histopathological examination of the spinal cord revealed that the number of neurons was preserved (Fig. 3B), and that infiltration of CD68‐positive macrophage/microglia (Fig. 3C). In contrast to colonic submucosal plexuses, the DRG displayed severe lymphocytic infiltration around the neurons (Fig. 3D). Infiltrating inflammatory cells were positive for CD8 (Fig. 3E) and CD68 (Fig. 3F), and negative for CD4 (Fig. 3G) and CD20 (Fig. 3H). We also observed his sciatic and sural nerves by using Epon‐embedded, toluidine blue‐stained semithin sections. In the left sciatic nerve, myelinated fibers showed no significant decrease in the density (Fig. 3I, J). In the sural nerve, the density of myelinated fibers slightly decreased (Fig. 3K), and some fibers were thinly myelinated (Fig. 3L). We also examined his left fibularis brevis muscle by using frozen sections. In the muscle, intramuscular nerve bundles were preserved (Fig. 3M, N), and cytoplasmic bodies were scattered on modified Gomori trichrome‐stained sections (Fig. 3N). Inflammatory cell infiltration was not observed in the muscle (Fig. 3 M–O). NADH‐tetrazolium reductase (TR) staining revealed that intermyofibrillar networks were preserved in the muscle (Fig. 3O).

## ASSESSMENT OF THE FREQUENCY OF PERIPHERAL NEUROPATHIES

To review the frequency of peripheral neuropathies, including Guillain‐Barré syndrome (GBS), in a larger population, we interrogated drug safety information provided by Merck & Co., Ono Pharmaceutical, and Bristol‐Myers Squibb, such as previous randomized trials of anti‐PD‐1 and anti‐CTLA‐4 antibodies. Drug information reported 66 events of peripheral neuropathies, including GBS as an irAE among 5122 patients (1.29%) until October 2019. Although there was no significant difference in the incidence of irAEs in other organs between anti‐PD‐1 and CTLA‐4 antibodies, patients treated with anti‐PD‐1 antibodies appeared to have more frequent and severe peripheral neuropathies compared to those who received anti‐CTLA‐4 antibody (1.59% vs. 0.69%; Fisher exact test, *P* < 0.001; three severe events vs. zero severe events) (Table 1). Pembrolizumab and nivolumab did not differ in the incidence of peripheral neuropathies, including severe neuropathies (grades 3–4).

**Table 1:** The incidences of peripheral neuropathy in patients receiving immune‐checkpoint blockades

Characteristics		Anti‐PD‐1 antibody		Anti‐CTLA‐4 antibody	*P*‐value
	All cases, *n* = 3962	Pembrolizumab, *n* = 2266	Nivolumab, *n* = 1696	Ipilimumab, *n* = 1160	
Any grade	63 (1.59%)	42 (1.85%)	21 (1.24%)	8 (0.69%)	*P* < 0.01
Grade ≥ 3	3 (0.08%)	1 (0.04%)	2 (0.12%)	0 (0.00%)	n.a.

n.a., not accessed.

## DISCUSSION

The irAEs most commonly affect the gastrointestinal tract, endocrine glands, skin, and liver, and they are thought to arise from aberrant activation of autoreactive T lymphocytes,^3^, ^4^ being more frequent and severe in the treatment with a combination of ipilimumab and nivolumab.^5^ On the other hand, phase II and III clinical trials reported that irAEs involving the central or peripheral nervous system are exceedingly rare. However, occasional neurological irAEs sometimes cause severe neurological disorders. In addition, compared to anti‐CTLA‐4 antibodies, anti‐PD‐1 antibodies have a higher risk of irAEs, especially severe in the peripheral nervous system, although there was no difference in the incidence of irAEs affecting these organs between anti‐PD‐1 antibodies and anti‐CTLA‐4 antibodies. The pathomechanism of neurological irAEs is still unclear and deserves particular attention.

Our patient presented subacute muscle weakness, dysesthesia, and colitis after receiving pembrolizumab. Subacute dysesthesia was one of the major symptoms of sensory neuronopathy, which is a subtype of paraneoplastic neurological disorders and is sometimes associated with anti‐Hu and anti‐Yo autoantibodies.^7^ Muscle weakness is often observed in patients with paraneoplastic subacute sensory neuronopathy. Subacute sensory neuronopathy with muscle weakness is called paraneoplastic sensorimotor neuronopathy, which is also classified in another subtype of paraneoplastic neurological disorders.^7^ Paraneoplastic sensorimotor and subacute sensory neuronopathies have been described as neurological syndromes characterized by primary neuronal cell body damage that involves not only sensory and motor nerves but also the peripheral autonomic nervous system. The pathological examination of our patient revealed the CD8‐positive lymphocytic infiltrates in DRG. This is consistent with the pathological findings described in case reports of paraneoplastic sensory neuronopathy.^8^, ^9^, ^10^ However, anti‐neuronal autoantibodies against Hu and Yo and anti‐ganglioside antibodies, such as GQ1b, were not detected in the serum.

PD‐1 and CTLA‐4 work as negative regulators of the priming and activation period in the cancer‐immunity cycle.^11^ In addition, PD‐1 acts as an inhibitor of the killing process in the cancer cell period of the cancer‐immunity cycle.^11^ Killing of the cancer cell induces the release of additional tumor‐associated antigens to increase the breadth and depth of the response in subsequent revolutions of the cycle.^11^ In our case, the surgical specimens indicative of transmural colitis demonstrated nonspecific inflammation with infiltration of both CD4‐ and CD8‐positive lymphocytes that did not infiltrate to the colonic plexuses. However, the postmortem examination in our case revealed that CD8‐positive lymphocytes infiltrated around neurons in the DRG in spite of the absence of serum anti‐neuronal autoantibodies against Hu and Yo, and antibodies against gangliosides, such as GQ1b. The pathological findings indicative of paraneoplastic neuronopathy suggest that tumor antigens and DRG have slight cross‐reactivity, and that pembrolizumab upregulates the response of cytotoxic T cells to DRG by activating of the killing of the cancer cell period. Further studies are needed to elucidate the pathomechanism of irAEs.

In this case, we reported a patient presenting subacute muscle weakness and dysesthesia with colitis as an irAE caused by pembrolizumab. The postmortem examination revealed polyradiculoneuropathy with CD8‐positive lymphocytic infiltration around neurons of the DRG and spinal cord, and it was consistent with pathological findings characteristic of paraneoplastic sensorimotor neuronopathy. These findings may contribute to elucidating the pathomechanism of irAEs in the nervous system.

## DISCLOSURE

The authors declare no conflicts of interest.
